# Increased Serum Levels of Fetal Tenascin-C Variants in Patients with Pulmonary Hypertension: Novel Biomarkers Reflecting Vascular Remodeling and Right Ventricular Dysfunction?

**DOI:** 10.3390/ijms18112371

**Published:** 2017-11-08

**Authors:** Ilonka Rohm, Katja Grün, Linda Marleen Müller, Daniel Kretzschmar, Michael Fritzenwanger, Atilla Yilmaz, Alexander Lauten, Christian Jung, P. Christian Schulze, Alexander Berndt, Marcus Franz

**Affiliations:** 1 Department of Internal Medicine I, Division of Cardiology, Angiology, Pneumology and Intensive Medical Care, Jena University Hospital, Friedrich-Schiller-University, 07747 Jena, Germany; Ilonka.Rohm@med.uni-jena.de (I.R.); Katja.Gruen@med.uni-jena.de (K.G.); Linda.Mueller@med.uni-jena.de (L.M.M.); DANIEL.KRETZSCHMAR@med.uni-jena.de (D.K.); Michael.Fritzenwanger@med.uni-jena.de (M.F.); Christian.Schulze@med.uni-jena.de (P.C.S.); 2 Department of Internal Medicine II, Division of Cardiology, Elisabeth Klinikum Schmalkalden, 98574 Schmalkalden, Germany; Atilla.Yilmaz@elisabeth-klinikum.de; 3 Department of Cardiology, Charité–Universitätsmedizin Berlin, 12203 Berlin, Germany; Alexander.Lauten@charite.de; 4 Department of Internal Medicine, Division of Cardiology, University Hospital Düsseldorf, Heinrich Heine University, 40225 Düsseldorf, Germany; christian.jung@med.uni-duesseldorf.de; 5 Institute of Pathology, Jena University Hospital, Friedrich-Schiller-University, 07743 Jena, Germany; Alexander.Berndt@med.uni-jena.de

**Keywords:** fetal tenascin-C, pulmonary hypertension, vascular remodeling, right ventricular dysfunction

## Abstract

Pulmonary vascular remodeling is a pathophysiological feature that common to all classes of pulmonary hypertension (PH) and right ventricular dysfunction, which is the major prognosis-limiting factor. Vascular, as well as cardiac tissue remodeling are associated with a re-expression of fetal variants of cellular adhesion proteins, including tenascin-C (Tn-C). We analyzed circulating levels of the fetal Tn-C splicing variants B^+^ and C^+^ Tn-C in serum of PH patients to evaluate their potential as novel biomarkers reflecting vascular remodeling and right ventricular dysfunction. Serum concentrations of B^+^ and C^+^ Tn-C were determined in 80 PH patients and were compared to 40 healthy controls by enzyme-linked immunosorbent assay. Clinical, laboratory, echocardiographic, and functional data were correlated with Tn-C levels. Serum concentrations of both Tn-C variants were significantly elevated in patients with PH (*p* < 0.05). Significant correlations could be observed between Tn-C and echocardiographic parameters, including systolic pulmonary artery pressure (B^+^ Tn-C: *r* = 0.31, *p* < 0.001, C^+^ Tn-C: *r* = 0.26, *p* = 0.006) and right atrial area (B^+^ Tn-C: *r* = 0.46, *p* < 0.001, C^+^ Tn-C: *r* = 0.49, *p* < 0.001), and laboratory values like BNP (B^+^ Tn-C: *r* = 0.45, *p* < 0.001, C^+^ Tn-C: *r* = 0.42, *p* < 0.001). An inverse correlation was observed between Tn-C variants and 6-minute walk distance as a functional parameter (B^+^ Tn-C: *r* = −0.54, *p* < 0.001, C^+^ Tn-C: *r* = −0.43, *p* < 0.001). In a multivariate analysis, B^+^ Tn-C, but not C^+^ Tn-C, was found to be an independent predictor of pulmonary hypertension. Both fetal Tn-C variants may represent novel biomarkers that are capable of estimating both pulmonary vascular remodeling and right ventricular load. The potential beneficial impact of Tn-C variants for risk stratification in patients with PH needs further investigation.

## 1. Introduction

Pulmonary hypertension (PH) is a clinical entity consisting of different conditions with elevated mean pulmonary arterial pressure (PAP) that is above 25 mmHg and is quantified by invasive measurements [1]. Elevation of PAP is associated with increased morbidity and mortality rates [2]. According to the classification used by current guidelines [1], five entities of PH are distinguished according to etiology. Group 1, pulmonary arterial hypertension (PAH), mainly includes the idiopathic and hereditary forms, as well as PH that is associated with connective tissue disease and drug intake. Group II is defined as PH due to left heart disease. Group III covers PH due to lung diseases and/or hypoxia. Group IV includes those conditions where chronic thromboembolic pulmonary events result in PH (CTEPH). Lastly, Group V covers miscellaneous disorders that lead to an increase in the pulmonary arterial pressure. However, significant overlap in clinical features and etiology exists [1].

Despite the diversity in PH etiology, certain pathophysiological processes occur in all forms of pulmonary hypertension. These include vasoconstriction, microthrombi formation, and pulmonary vascular remodeling, including structural and functional rebuilding of the extracellular matrix (ECM) [3]. Pathological tissue remodeling is known to be accompanied by the re-occurrence of fetal variants of matrix proteins, like tenascin-C (Tn-C), which are absent in non-diseased mature tissues. This effect has been repeatedly demonstrated for remodeling of cardiac tissue [4,5,6,7,8,9], but also occurs during vascular remodeling [10]. The cell adhesion molecule Tn-C is known to play a pivotal role in the regulation of cell adhesion, activation, differentiation, and migration in inflammation and tissue remodeling [11]. Different so-called fetal variants of this protein are generated by alternative splicing of the pre-mRNA, in particular B and C-domain, containing Tn-C (B^+^ and C^+^ Tn-C) [12,13]. These variants are expressed during embryonic development and under pathological conditions, while being virtually absent in healthy adult organs [14].

Because of the close association of fetal Tn-C variants and tissue, as well as vascular remodeling, the aim of the present study was to investigate the serum concentrations of B^+^ and C^+^ Tn-C in patients with pulmonary hypertension as compared to healthy control persons. Additionally, the assessment of echocardiographic, standard laboratory and functional parameters were performed to enable correlation analyses with circulating Tn-C to elucidate the possible role of these protein variants as novel biomarkers for diagnosis and risk stratification in patients with PH.

## 2. Results

### 2.1. Baseline Characteristics

For these investigations, 80 patients with PH and 40 apparently healthy subjects were recruited. Baseline characteristics of the included PH patients, as well as controls are reported in Table 1. PH patients had more co-morbidities, such as hyperlipidemia, coronary artery disease, diabetes mellitus, and chronic kidney disease. Medical therapy of the PH patients included more frequent intake of statins and glucocorticoids. Laboratory analyses revealed higher BNP, CRP, and creatinine, and lower LDL cholesterol and hemoglobin. The lower LDL cholesterol is at least in part explained by the higher intake of statins in the PH patients but also the chronic inflammatory state.

### 2.2. Serum Levels of B^+^ and C^+^ Tn-C

**Figure 1:**
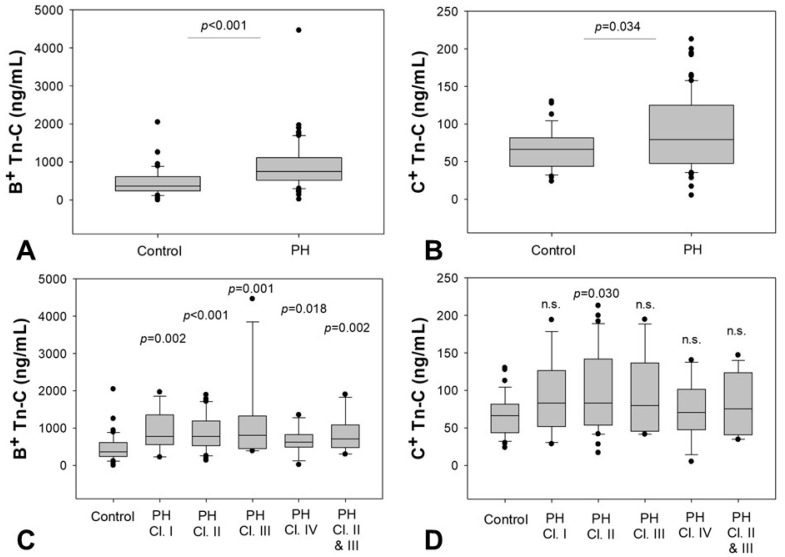
Increased serum levels of B^+^ and C^+^ Tn-C in patients with pulmonary hypertension (PH) compared to healthy controls. (**A**) shows the results for B^+^ Tn-C; (**B**) for C^+^ Tn-C; (**C**,**D**) show the subgroup analyses with respect to the PH etiology, PH classes are named according to the classification of the current guidelines. The box plots indicate the median (line inside the box), 25 and 75 percentile (lower and upper boundary of the box), 10 and 90 percentile (whiskers outside the box) as well as outlier values (dots). Cl. = class, Tn-C = Tenascin C.

Both fetal splicing variants of Tn-C were higher in patients with PH (B^+^ Tn-C: 752 (CI 520–1110) ng/mL versus 368 (CI 238–616) ng/mL, *p* < 0.001, Figure 1A; C^+^ Tn-C: 79 (CI 48–125) ng/mL versus 66 (CI 44–82) ng/mL, *p* = 0.034, Figure 1B).

For B^+^ Tn-C, analysis with respect to the PH etiology revealed significant elevations compared to healthy controls for all classes investigated: class I (780 (CI 559–1350) ng/mL, *p* = 0.002), class II (778 (CI 530–1191) ng/mL, *p* < 0.001), class III PH (811 (CI 447–1324) ng/mL, *p* = 0.001), and class IV (624 (CI 489–829) ng/mL, *p* = 0.018). Similar results were found for PH of mixed genesis, including PH resulting from lung and cardiac diseases (class II and class III) (706 (CI 478–1089) ng/mL, *p* = 0.002) (Figure 1C). For C^+^ Tn-C, significantly elevated concentrations were observed in patients with PH when compared to controls, but subgroup analysis only revealed a significant difference in patients with PH class II as compared to healthy controls (PH class II: 83 (CI 54–142), *p* = 0.030) (Figure 1D).

### 2.3. Correlation of Tn-C Serum Levels with Echocardiographic Parameters

**Figure 2:**
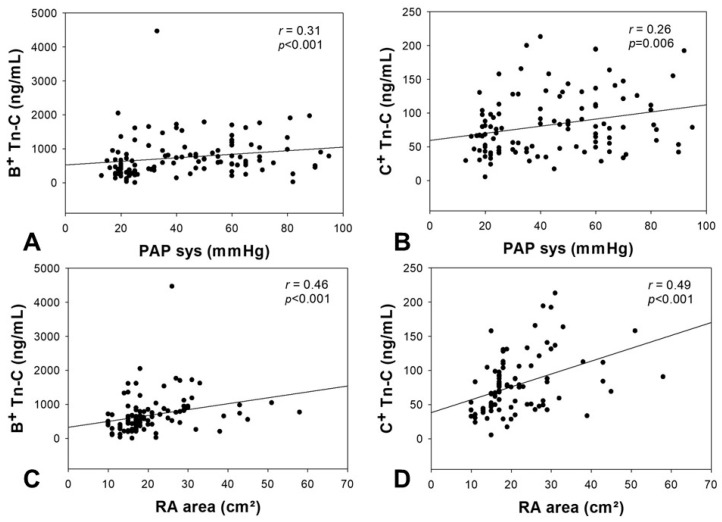
Correlation analyses between the serum concentration of B^+^ and C^+^ Tn-C and echocardiographic parameters. The correlation analysis graphs demonstrate significant correlations between B^+^ and C^+^ Tn-C and the systolic pulmonary artery pressure (**A**,**B**), as well as the area of the right atrium (**C**,**D**). PAP sys = systolic pulmonary arterial pressure, RA = right atrium, Tn-C = tenascin C, *p*-value = level of significance, r-value = correlation coefficient.

For both patients’ groups, echocardiographic parameters were obtained (Table 2) and were correlated with the serum concentrations of Tn-C. For both variants of Tn-C, a significant correlation between the serum concentration and the systolic PAP was observed (B^+^ Tn-C: *r* = 0.31, *p* < 0.001, C^+^ Tn-C: *r* = 0.26, *p* = 0.006). Additionally, a significant correlation between the serum levels of B^+^ and C^+^ Tn-C and the right atrial area was found (B^+^ Tn-C: *r* = 0.46, *p* < 0.001, C^+^ Tn-C: *r* = 0.49, *p* < 0.001). These correlations are demonstrated in Figure 2. No significant correlation was observed between Tn-C variants and TAPSE or TDI of the RV as markers of right heart dysfunction.

### 2.4. Correlation of Tn-C Serum Levels with BNP

**Figure 3:**
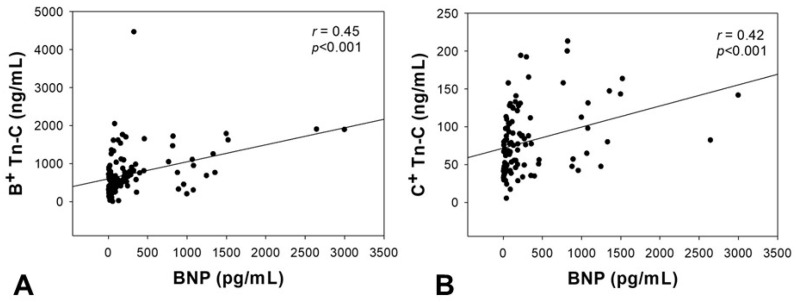
Correlation analyses between the serum concentration of B^+^ (**A**) and C^+^ (**B**) Tn-C and the brain natriuretic peptide (BNP). BNP = brain natriuretic peptide, Tn-C = tenascin C, *p*-value = level of significance, *r*-value = correlation coefficient.

Serum concentration of Tn-C correlated with BNP as a standard laboratory parameter of heart failure. For both Tn-C variants, a significant correlation could be demonstrated (B^+^ Tn-C: *r* = 0.45, *p* < 0.001; C^+^ Tn-C: *r* = 0.42, *p* < 0.001, Figure 3).

### 2.5. Correlation of Tn-C Serum Concentrations with the 6-Minute Walk Distance

**Figure 4:**
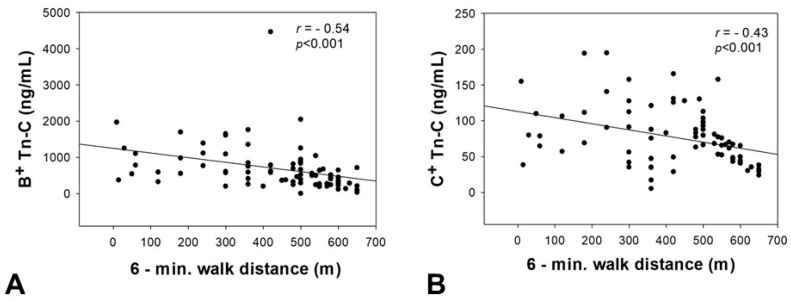
Correlation analyses between the serum concentration of B^+^ (**A**) and C^+^ (**B**) Tn-C and the 6-min walk distance. Tn-C = tenascin C, *p*-value = level of significance, *r*-value = correlation coefficient.

An inverse correlation was observed between serum levels of the two Tn-C variants and the 6-minute walk distance (B^+^ Tn-C: *r* = −0.54, *p* < 0.001; C^+^ Tn-C: *r* = −0.43, *p* < 0.001) as a parameter reflecting functional capacity (Figure 4).

### 2.6. Impact of Clinical Parameters on Tn-C Serum Levels

Because of significant clinical differences in the patient groups that were enrolled in our study, we performed further analyses investigating possible confounding variables. To verify the predictive value of Tn-C serum concentration for the probability of pulmonary hypertension, a multivariate analysis was performed. Age, diabetes mellitus, CAD, CKD, statin medication, C-reactive protein levels, and the serum concentration of the two Tn-C variants were entered into the analysis as independent variables. After backward elimination, only CKD (Wald: 9.529, OR: 12.316, 95% CI: 2.501–60.647, *p* = 0.002), C-reactive protein (Wald: 4.414, OR: 1.199, 95% CI: 1.012–1.420, *p* = 0.036), and B^+^ Tn-C (Wald: 7.854, OR: 1.002, 95% CI: 1.001–1.004, *p* = 0.005), but not age, diabetes mellitus, CAD, statin medication, and C^+^ Tn-C were found to be independent predictors of pulmonary hypertension.

## 3. Discussion

Pathological tissue and vascular remodeling is associated with a variety of cardiovascular disorders results in the re-occurrence of fetal variants of extracellular matrix and cell adhesion modulating proteins like Tn-C, which are virtually absent in non-diseased adult organs [14]. The re-expression of fetal Tn-C variants like B^+^ and T^+^ Tn-C was repeatedly demonstrated to reflect the extent of cardiovascular remodeling and disease severity for several heart diseases [6,7,9]. Therefore, these molecules have been suggested as novel biomarkers not only for diagnosis and prognosis estimation, but also for therapeutic surveillance since serum levels also reflect reverse remodeling [4,5,6,9,15,16]. Fetal Tn-C re-occurrence has also been demonstrated with special regard to vascular remodeling processes [17,18].

In an animal model using monocrotaline-injected rats, Rabinovitch and colleagues demonstrated that the development of pulmonary arterial medial hypertrophy was accompanied by Tn-C expression in co-localization with proliferating smooth muscle cells [17]. Additionally, it could be shown that iloprost inhalation results in a reversion of the remodeling process reflected by a decrease in Tn-C occurrence [18]. Furthermore, Correira-Pinto et al. also demonstrated an overexpression of Tn-C in cardiac tissue of monocrotaline-induced pulmonary hypertension in an animal model reflecting PH-induced cardiac, and in particular, right ventricular pressure overload [19]. However, these observations have only been made for Tn-C found in tissue, not serum, and it is well known that lung tissue from PH patients is not available in daily routine because taking biopsies is not indicated in these patients and will not be an option, even in the future, due to the high risk associated with this invasive procedure.

In the present study, we investigated fetal Tn-C variants as serum biomarkers of tissue and vascular remodeling in human PH. We demonstrate that fetal Tn-C levels are increased in PH when compared to healthy controls. Elevated Tn-C concentrations in patients suffering from PH have previously been described by Schumann et al. [20]. The authors, however, did not analyze different fetal Tn-C variants [20]. Therefore, our data are the first to demonstrate both B^+^ and C^+^ Tn-C as functionally most important fetal splicing variants of the protein with prognostic significance in PH.

Subgroup analyses of the present study revealed that an elevated serum concentration of B^+^ Tn-C occurs in PH due to different etiologies as compared to healthy controls. For C^+^ Tn-C, subgroup analysis revealed significant differences only between PH class II and the control group. It has to be mentioned, that in this PH class, the highest number of patients has been included. Besides this, the intake of glucocorticoids might diminish statistical significance in PH due to lung diseases. An influence of glucocorticoids on Tn-C expression has been previously described in the literature [21,22,23].

Interestingly, Tn-C serum concentrations could be shown to correlate with echocardiographic parameters, such as systolic PAP and the area of the right atrium. This demonstrates significant correlations between the fetal variants of the matrix glycoprotein and parameters reflecting right ventricular load. This finding is supported by former studies that found PH-induced cardiac remodeling with an increased Tn-C occurrence [19]. This idea is supported by a publication investigating a patient cohort with acute pulmonary thromboembolism describing a correlation between Tn-C and systolic PAP [24]. Additionally, similar effects have been described in an animal model. Monocrotaline-induced right ventricular failure was shown to be associated with an up-regulation of Tn-C gene expression and results in significantly elevated plasma levels. However, in this model, a significant correlation could be observed for right ventricular ejection fraction and Tn-C [25]. In our present study, as markers of right ventricular function, TAPSE and TDI were echocardiographically measured but no significant correlation between these parameters and Tn-C were observed.

Another interesting finding of the present study was a significant positive correlation between serum BNP and both, B^+^ and C^+^ Tn-C levels. This reflects prior findings in human studies of patients with acute PH due to pulmonary thromboembolism [24], as well as animal models [25]. Besides PH, this correlation has also repeatedly been described in other cardiovascular diseases [16]. However, the present study was not only able to demonstrate a correlation between the fetal Tn-C variants and laboratory parameters, but also with functional data reflecting the physical capability of PH patients assessed by the 6-min walk test. This association was found for both Tn-C variants and is a novel finding of the current study.

There are certain limitations of our study. First, due to the small numbers of patients, statistical analyses underestimate the differences. Second, heterogeneity with respect to the clinical data of PH patients and the controls has to be mentioned. Moreover, significant differences in the subgroup analyses can be explained by the PH etiology. The highest mean BNP value is found in the PH class II, reflecting PH due to left heart disease. This is possibly due to the increased cardiac load not only of the right heart, but also the left heart occurring in the course of the underlying disease.

## 4. Material and Methods

### 4.1. Patients

We enrolled 80 patients with PH admitted to the Department of Internal Medicine I, Jena University Hospital, Friedrich Schiller University of Jena, Germany. 40 apparently healthy control subjects were recruited within the same department. Controls were enrolled after invasive exclusion of coronary artery disease [26]. Further exclusion criteria were malignant or autoimmune disease, hyperthyroidism, infection, history of pulmonary embolism or stroke, peripheral artery disease, and medical treatment, including corticosteroids or immunosuppressive agents. All of the patients underwent transthoracic echocardiography and a 6-minute walk test. Blood samples were collected for routine laboratory analyses, including serum brain natriuretic peptide (BNP) levels. Serum was stored using special low binding tubes (Protein LoBind Tubes, Eppendorf AG, Hamburg, Germany) and stored at −80 °C after snap freezing in liquid nitrogen to reduce artificial protein degradation. Moreover, repeated freeze-thaw cycles were strictly avoided.

The investigation conforms with the principles outlined in the *Declaration of Helsinki* (*Br Med J 1964*; ii: 177) and was approved by the local ethics committee (registration number: 4732-03/16, 11 April 2016). All patients gave written informed consent before inclusion into the study.

### 4.2. Quantification of Serum Tn-C Levels and Standard Laboratory Values

Serum levels of B^+^ and C^+^ Tn-C were determined using enzyme-linked immunosorbent assay (ELISA). Well-established and validated ELISA assays are commercially available (Tenascin-C Large (FNIII-C) ELISA and Tenascin C Large (FNIII-B) ELISA, both IBL International GmbH, Hamburg, Germany). Routine standard laboratory parameters were measured according to standard hospital procedures.

### 4.3. Investigation of Echocardiographic Parameters and 6-Minute Walk Test

For each patient, a transthoracic echocardiography was performed to determine standard parameters, including left ventricular ejection fraction (EF), diastolic diameter of the interventricular septum (IVSDd), or relevant valve abnormalities. Moreover, special effort was made to carefully assess the parameters representing right heart morphology and dysfunction. Here, especially tricuspid annular plane systolic excursion (TAPSE), tissue doppler imaging of the right ventricle (TDI RV), the area of the right atrium (RA), and systolic pulmonary arterial pressure were measured. Moreover, 6-minute walk distance was documented for each patient.

### 4.4. Statistical Analysis

Statistical analysis was performed using SPSS (version 20.0, IBM Inc., Armonk, NY, USA) and SigmaPlot (version 12.0, Systat Software Inc., San Jose, CA, USA). The Kolmogorov Smirnov test was used to test the normal distribution of all the variables. When normally distributed, values are reported as mean ± standard deviation. When not normally distributed, the values are reported as median (25–75% Confidence Interval). The non-parametric Mann-Whitney Rank Sum Test was used to compare the number of different cells between two different study groups. To compare more than two groups, the Kruskal Wallis test was used. Bivariate correlations between parametric variables were assessed by the Spearman rank correlation test. To test the predictive value of Tn-C concentrations on the probability of the occurrence of pulmonary hypertension, a multivariate regression analysis was performed using a binary logistic model (backward elimination method: Wald). The presence of pulmonary hypertension was defined as the dependent variable. Age, diabetes mellitus, CAD, CKD, C-reactive protein, statin treatment, and the two Tn-C variants were included into the first step. Then, multistep backward elimination (removal threshold *p* > 0.10) of independent variables was carried out. *p* < 0.05 was considered statistically significant.

## 5. Conclusions

In conclusion, the present study demonstrates that there is an increase of serum of B^+^ and C^+^ Tn-C in patients with PH. For B^+^ Tn-C, this is visible for all PH classes. This is an important novel finding, because most of the studies in the field of PH have been conducted in PAH, which is a rare disease [27]. The prevalence of especially class II and class III PH is more frequent. For this reason, research in this field is necessary to improve diagnostic and therapeutic approaches.

Based on our findings, both the investigated Tn-C splicing variants, but especially B^+^ Tn-C, can be suggested as promising novel biomarkers in human PH, which merits further investigation and validation in larger patient cohorts. Fetal Tn-C variants are functionally involved in vascular and tissue remodeling associated with PH probably irrespective of the particular etiology. This should be further investigated both in vivo and in vitro and raises the question, whether functional blocking strategies might be a therapeutic approach for PH treatment in the future. In this context, the availability of human recombinant antibodies specific to fetal Tn-C variants might be of certain interest since these antibodies can serve as vehicles for targeted delivery of both, bioactive molecules, such as immunocytokines and antibody-drug-conjugates. Further, this might have diagnostic potential for the development of novel radionuclides or molecular imaging strategies [28,29,30] to visualize pathologic lung tissue and vascular remodeling.

## Figures and Tables

**Table:** (**A**)

Clinical Parameter	Control Persons (*n* = 40)	PH Patients (*n* = 80)	*p*-Value
Age (years)	66 ± 7	70 ± 13	n.s.
BMI (kg/m^2^)	27.9 ± 4.7	28.6 ± 6.0	n.s.
Gender, male (%)	33	39	n.s.
Systolic BP (mmHg)	146 ± 33	146 ± 33	n.s.
Diastolic BP (mmHg)	81 ± 17	78 ± 13	n.s.
Functional class	1.5 ± 0.6	2.6 ± 0.8	<0.001
**Laboratory:**			
BNP (pg/mL)	56 ± 70	445 ± 584	<0.001
CRP (mg/L)	2.7 ± 2.6	11.7 ± 17.5	0.002
Creatinine (μmol/l)	76 ± 16	111 ± 50	<0.001
LDL (mmol/l)	3.6 ± 1.0	2.7 ± 1.0	<0.001
Haemoglobin (mmol/l)	8.7 ± 0.8	7.9 ± 1.3	<0.001
Leukocytes (Gpt/l)	7.1 ± 1.4	7.5 ± 2.2	n.s.
**Co-Morbidities:**			
Hypertension (%)	95	83	0.03
Hyperlipidemia (%)	88	56	<0.001
Obesity (%) (BMI>30 kg/m^2^)	42	38	n.s.
CAD (%)	0	26	<0.001
CKD (%) (GFR < 50 mL/min)	8	50	<0.001
Diabetes (%)	20	50	0.002
Smoking (%)	29	52	0.027
**Medication:**			
ASA (%)	25	18	n.s.
Beta blocker (%)	63	60	n.s.
ACE Inhibitor/Sartans (%)	85	78	n.s.
Statins (%)	38	59	0.026
Prednisolon (%)	0	11	0.027
ICS (%)	0	21	0.002

**Table:** (**B**)

Characteristics	Control (*n* = 40)	PH Class I (*n* = 13)	PH Class II (*n* = 30)	PH Class III (*n* = 11)	PH Class IV (*n* = 12)	PH Class II & III (*n* = 14)	*p*-Value between Different PH-Classes
Age (years)	66.0 ± 6.8	64.5 ± 11.5	75.1 ± 8.1	58.5 ± 22.3	69.8 ± 11.5	74.9 ± 7.9	<0.001
BMI (kg/m^2^)	28.0 ± 4.7	29.0 ± 8.4	27.9 ± 3.9	25.9 ± 7.5	30.6 ± 5.2	29.9 ± 6.5	n.s.
Functional class	1.5 ± 0.6	2.6 ± 0.8	2.6 ± 0.7	2.4 ± 1.1	2.3 ± 0.7	2.8 ± 0.7	n.s.
**Laboratory:**							
BNP (pg/mL)	56 ± 70	113 ± 82	635 ± 678	469 ± 469	111 ± 87	627 ± 731	0.021
CRP (mg/L)	2.7 ± 2.6	7.8 ± 10.7	13.8 ± 21.0	10.5 ± 12.9	5.2 ± 5.8	16.1 ± 22.0	n.s.
Creatinine (μmol/L)	76 ± 16	88 ± 44	127 ± 59	93 ± 24	99 ± 40	121 ± 47	n.s.
LDL (mmol/L)	3.6 ± 1.0	2.5 ± 1.0	2.5 ± 1.0	3.0 ± 1.0	3.0 ± 0.9	2.7 ± 1.2	n.s.
Haemoglobin (mmol/L)	8.7 ± 0.8	7.7 ± 1.5	7.5 ± 1.2	8.5 ± 1.0	8.9 ± 1.4	7.6 ± 1.6	0.012
Leukocytes (Gpt/L)	7.1 ± 1.4	6.7 ± 2.4	7.6 ± 1.7	8.3 ± 1.5	7.4 ± 1.8	7.7 ± 3.4	n.s.

Data are presented as mean ± standard deviation or percentage. ACE = angiotensin-converting enzyme, ASA = acetyl-salicylic acid, BMI = Body mass index, BNP—brain natriuretic peptide, CHD = coronary heart disease, CKD = chronic kidney disease, CRP = C-reactive protein, LDL = low-density lipoprotein, n.s. = not significant.

**Table 2:** Echocardiographic parameters of the study groups.

Parameter	Control (*n* = 40)	PH Class I (*n* = 13)	PH Class II (*n* = 30)	PH Class III (*n* = 11)	PH Class IV (*n* = 12)	PH Class II & III (*n* = 14)
LV-EF (%)	68 ± 7	63 ± 7	56 ± 13	63 ± 9	60 ± 6	52 ± 12
IVSDd (mm)	12 ± 2	12 ± 3	13 ± 3	13 ± 2	11 ± 2	12 ± 2
RVd basal (mm)	35 ± 2	41 ± 9	46 ± 7	47 ± 10	44 ± 7	49 ± 9
TAPSE (mm)	25 ± 2	22 ± 5	15 ± 3	19 ± 7	18 ± 3	16 ± 4
TDI-S’ (RV) (cm/s)	14 ± 2	12 ± 1	8 ± 1	9 ± 0	11 ± 1	9 ± 2
RA area (cm^2^)	15.4 ± 2.3	20.8 ± 8.3	27.9 ± 9.6	19.7 ± 8.8	22.2 ± 5.2	31.0 ± 13.7
PAP sys (mmHg)	21 ± 4	59 ± 22	52 ± 16	57 ± 22	50 ± 20	56 ± 16

Data are presented as mean ± standard deviation. IVSDd = intraventricular septum diameter in diastole, LV-EF = left ventricular ejection fraction, PAP sys = systolic pulmonary arterial pressure, RA = right atrium, RVd = right ventricular diameter in diastole, TAPSE = tricuspid annular plane systolic excursion, TDI-S’ (RV) = tissue doppler imaging, right ventricle. PH classes given in this table correspond to the PH groups defined in the current guidelines.

## Abbreviations

ACE	Angiotensin-converting enzyme
ASA	Acetyl-salicylic acid
BMI	Body mass index
BNP	Brain natriuretic peptide
CAD	Coronary artery disease
CKD	Chronic kidney disease
CRP	C-reactive protein
ECM	Extracellular matrix
IVSDd	Intraventricular septum diameter in diastole
LDL	Low-density lipoprotein
LV-EF	Left ventricular ejection fraction
PH	Pulmonary hypertension
PAH	Pulmonary arterial hypertension
PAP	Pulmonary arterial pressure
RA	Right atrium
RV	Right ventricle
TAPSE	Tricuspid annular plane systolic excursion
TDI	Tissue doppler imaging
Tn-C	Tenascin C
